# Transcriptomic Insights into Anthocyanin Biosynthesis in *Aronia melanocarpa* Callus Under Different Light Conditions

**DOI:** 10.3390/ijms26199588

**Published:** 2025-10-01

**Authors:** Mingjun Hou, Bingrui Wang, Chang An, Yulai Wu, Mohammad Gul Arabzai, Xiaopeng Fan, Changbing Liu, Zongshen Zhang

**Affiliations:** 1 Laboratory of Pharmaceutical Plant Cell Engineering, School of Biological Engineering, Dalian Polytechnic University, Dalian 116034, China; 18609475232@163.com (M.H.); wuyulai0626@163.com (Y.W.); liucb@dlpu.edu.cn (C.L.); 2 Hangzhou Institute of Advanced Technology, Hangzhou 310018, China; xp.fan@hiat.ac.cn; 3 College of Plant Science & Technology, Huazhong Agricultural University, Wuhan 430070, China; brwang@mail.hzau.edu.cn; 4 College of Agriculture, Fujian Provincial Key Laboratory of Haixia Applied Plant Systems Biology, College of Life Sciences, Fujian Agriculture and Forestry University, Fuzhou 350002, China; arabzai27@gmail.com

**Keywords:** *Aronia melanocarpa*, callus culture, light quality, transcriptome analysis, anthocyanin

## Abstract

*Aronia melanocarpa* is rich in anthocyanins, compounds with significant medicinal and industrial value, making it an attractive species for enhanced production. Compared with fruits or intact plants, callus tissue offers a uniform, controllable in vitro system that is particularly suitable for dissecting regulatory mechanisms under defined environmental conditions. Although light quality is known to influence anthocyanin biosynthesis, its specific regulatory mechanisms in *A. melanocarpa* remain unclear. In this study, callus tissues were cultured under six light regimes: full-spectrum LED, blue:red (5:1), red:blue (5:1), red:blue:white (1:1:1), red:white (5:1), and pure blue light. Anthocyanin content was quantified using the pH differential method, and the results showed that the blue:red (5:1) treatment produced the highest accumulation, reaching 14.06 mg/100 g. Transcriptome sequencing was then performed to compare the gene expression profiles between calli cultured under blue:red (5:1) light and those maintained in darkness. A total of 10,547 differentially expressed genes (DEGs) were identified, including 6134 upregulated and 4413 downregulated genes. Functional enrichment analysis indicated that these DEGs were mainly involved in anthocyanin biosynthesis and transport. Importantly, key structural genes such as *PAL*, *C4H, 4CL*, *CHS*, *ANS*, *UFGT*, and *GST* were significantly upregulated under blue:red (5:1) light, as further validated by qRT-PCR. Overall, our findings demonstrate that a blue:red (5:1) light ratio enhances anthocyanin accumulation by promoting the expression of biosynthetic and transport-related genes. This study not only provides new transcriptomic insights into the light-mediated regulation of secondary metabolism in *A. melanocarpa* callus, but also establishes a foundation for optimizing in vitro culture systems for sustainable anthocyanin production.

## 1. Introduction

*Aronia melanocarpa*, also known as wild chokeberry or black chokeberry, a member of the genus Aronia in the family Rosaceae, is a perennial deciduous shrub native to the eastern regions of North America. It commonly grows in wetlands and forest margins from eastern Canada to the southeastern United States [1]. The plant is characterized by glossy foliage that turns red in autumn, white to pink flowers in late spring, and black-purple berries in late summer, making it valuable for both ornamental and economic purposes [2]. The fruits of *A. melanocarpa* are recognized for their exceptionally high content of polyphenolic compounds, among which anthocyanins predominate, making them one of the richest known dietary sources of these pigments [3]. Quantitative studies report that the total anthocyanin content in *A. melanocarpa* fruits ranges between 357 and 461 mg per 100 g fresh weight, and can reach 1000–2000 mg per 100 g dry weight during ripening. This extraordinary accumulation of anthocyanins underscores the remarkable medicinal value of *Aronia melanocarpa*.

The biosynthesis of anthocyanins is strongly influenced by environmental factors, especially light quality. Spectral composition, particularly the balance of blue and red wavelengths, has been shown to modulate anthocyanin accumulation across various fruit crops, including grape, cherry, blueberry, and apple, by regulating structural genes (*PAL*, *CHS*, *DFR*, *ANS*, *UFGT*) and transport-related proteins (*GST*) via photomorphogenic signaling pathways [4,5,6,7]. These findings highlight the central role of light as both a developmental signal and a metabolic regulator.

Compared to fruits and seedlings, however, the regulatory mechanisms of anthocyanin biosynthesis in callus tissues remain largely unexplored. Plant callus cultures provide a simplified yet powerful system for dissecting the environmental regulation of secondary metabolism, as they eliminate the complexity of whole-plant development while maintaining biosynthetic capacity [8]. The callus offers highly controllable experimental conditions, allowing the precise manipulation of light spectra and facilitating integrative analyses of metabolite accumulation and gene expression [9]. Furthermore, callus culture represents a promising biotechnological platform for the sustainable production of anthocyanins, with potential applications in pharmaceuticals and functional foods [10].

In this study, we employed *A. melanocarpa* callus as a model system to investigate the effects of different blue:red light ratios on anthocyanin biosynthesis and transport. By combining metabolite quantification, transcriptomic profiling, and qRT-PCR validation, we aimed to identify light-responsive genes (e.g., *PAL*, *C4H*, *4CL*, *CHS*, *ANS*, *UFGT*, *GST*) and elucidate the molecular framework underlying anthocyanin accumulation under controlled light conditions. Our findings provide novel insights into light-regulated secondary metabolism and support the use of callus cultures as a platform for optimizing anthocyanin production.

## 2. Results

### 2.1. Anthocyanin Synthesis in A. melanocarpa Under Different Light Conditions

**Figure 1:**
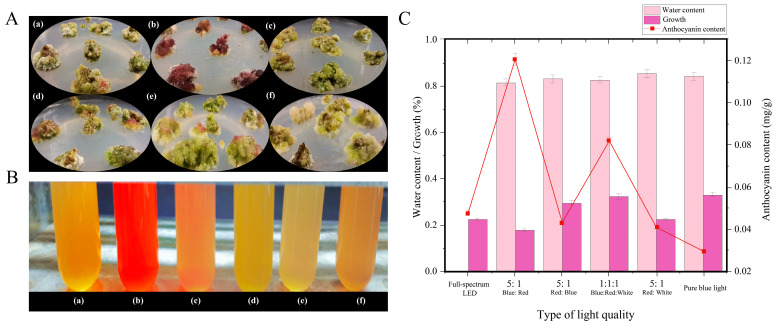
Effects of different light quality conditions on pigmentation, anthocyanin coloration, and growth of *A. melanocarpa* callus. (**A**) Pigmentation of callus tissue under different light treatments: (a), full-spectrum LED (light-emitting diode); (b), blue:red (5:1); (c), red:blue (5:1); (d), blue:red:white (1:1:1); (e), red:white (5:1); (f), pure blue light. (**B**) Anthocyanin coloration of callus extracts under acidic conditions from the corresponding treatments: (a), LED; (b), blue:red (5:1); (c), red: (5:1); (d), blue:red: white (1:1:1); (e), red:white (5:1); (f), pure blue light. (**C**) Water content, growth rate, and anthocyanin content of callus under different light treatments.

Calli of *A. melanocarpa* were cultured under six different light treatments: full-spectrum LED, blue:red (5:1), red:blue (5:1), red:blue:white (1:1:1), red:white (1:5), and monochromatic blue light. As shown in Figure 1, it was clearly observed that the callus tissue under blue:red (5:1) light exhibited a dark red pigmentation (Figure 1A), while other the spectra produced green tissues. We present the extraction of anthocyanins from cultured callus using an organic solvent-assisted ultrasonic extraction method (Figure 1B). The extracts from the blue:red (5:1) light treatment exhibited bright red coloration (indicative of high anthocyanin content under acidic conditions) (Figure 1B), while the other samples showed yellow-green hues. Quantitative analysis revealed significantly higher anthocyanin accumulation (14.06 mg/100 g) under blue:red (5:1) light compared to other spectral conditions (Figure 1C). Correspondingly, the callus in this treatment group (Sample B) exhibited both enhanced red pigmentation in extracts and increased biomass production.

### 2.2. Transcriptome Sequencing Analysis

RNA-seq analysis was conducted on *A. melanocarpa* callus exposed to blue:red (5:1) light (Group A) and dark conditions (Group B). The six libraries generated 36.10–57.76 Mb of raw reads. After quality control with Trimmomatic (v0.39) to remove adapter sequences and low-quality reads (Phred score < 20), we obtained 33.99–55.41 Mb high-quality clean reads per sample (Table 1). All samples exhibited excellent sequencing quality, with Q30 scores > 95.5% and GC content of 49.64–50.19%, fulfilling all the quality thresholds for downstream bioinformatics analyses.

### 2.3. Differential Gene Expression Analysis

**Figure 2:**
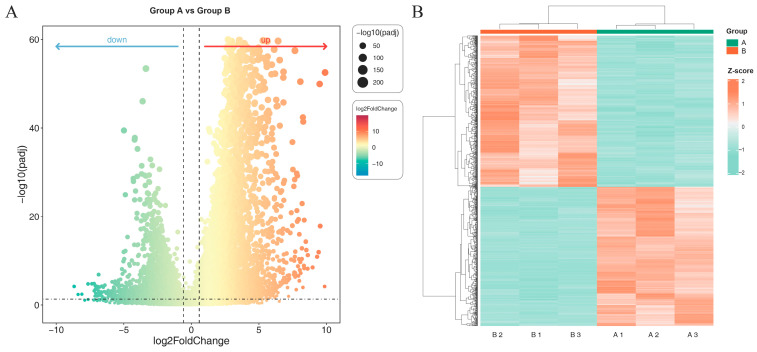
Differentially expressed genes (DEGs) visualized by (**A**) volcano plot and (**B**) hierarchical clustering heatmap. (**A**) Volcano plot showing DEGs between Group A and Group B. The x-axis represents the log2 fold change, and the y-axis shows the −log10 adjusted *p*-value (padj). Each dot corresponds to a gene, with significantly up-regulated genes (red, **right**) and down-regulated genes (green, **left**). The size of the dots is proportional to −log10(padj). (**B**) Heatmap of hierarchical clustering of DEGs based on Z-score normalized expression values. Rows represent genes and columns represent samples from Groups A and B. Red indicates higher expression levels, while green indicates lower expression levels.

Differential gene expression analysis was performed using DESeq2 (v1.38.3) on RNA-seq data from *A. melanocarpa* callus grown under blue:red (5:1) light (Group A) versus dark conditions (Group B). Applying stringent thresholds (|log2FC| ≥ 1.5, FDR < 0.05), we identified 10,547 differentially expressed genes (DEGs), comprising 6134 upregulated and 4413 downregulated genes in Group A relative to Group B (Figure 2A). Hierarchical clustering of these DEGs revealed clear separation between the light-treated and control groups, with high intra-group reproducibility among biological replicates (Figure 2B).

**Figure 3:**
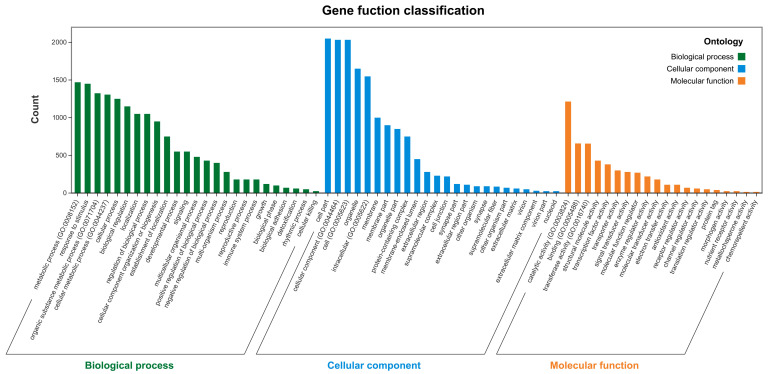
Gene Ontology (GO) functional classification of differentially expressed genes (DEGs). Bars are grouped into three categories: biological process (BP, green), cellular component (CC, blue), and molecular function (MF, orange). The x-axis shows representative GO terms, and the y-axis indicates the number of DEGs annotated to each term.

The Gene Ontology (GO) enrichment analysis of differentially expressed genes (DEGs) revealed significant enrichment across three functional categories (Figure 3): BP (biological process), CC (cellular component), and MF (molecular function). In the BP group, 1470 DEGs were annotated to metabolic process (GO:0008152), 1324 DEGs to organic substance metabolic process (GO:0071704), and 1306 DEGs to cellular metabolic process (GO:0044237). In the CC group, 2033 DEGs were annotated to cell (GO:0005623), 2033 DEGs to cellular component (GO:0044464), and 1548 DEGs to intracellular (GO:0005622). In the MF group, 1214 DEGs were annotated to catalytic activity (GO:0003824), 658 DEGs to binding (GO:0005488), and 656 DEGs to transferase activity (GO:0016740).

**Figure 4:**
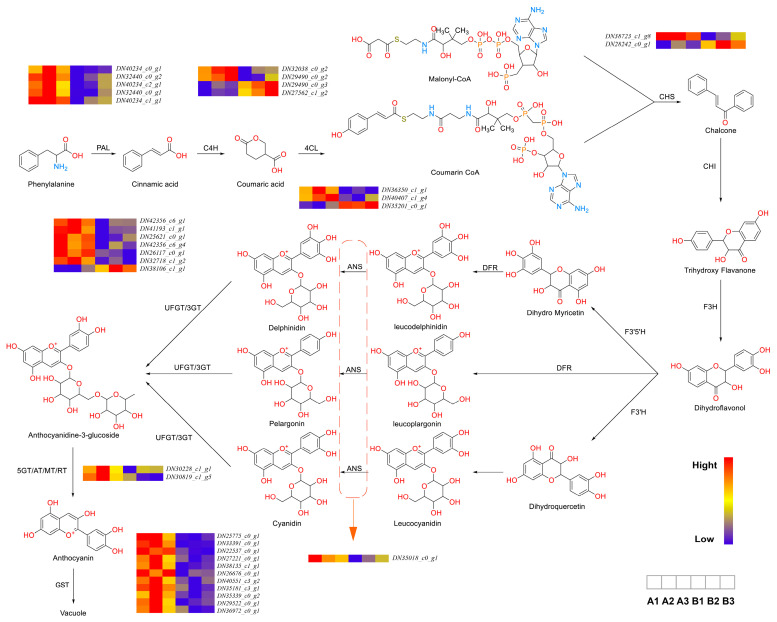
Enrichment analysis of KEGG pathway of genes related to Anthocyanidin (**left**) flavonoid metabolic pathway (**right**). The key enzymes involved in the pathway include PAL (phenylalanine ammonia-lyase), C4H (cinnamate 4-hydroxylase), 4CL (4-coumaroyl-CoA ligase), CHS (chalcone synthase), CHI (chalcone isomerase), F3H (flavanone 3-hydroxylase), F3′H (flavonoid 3′-hydroxylase), F3′5′H (flavonoid 3′,5′-hydroxylase), DFR (dihydroflavonol 4-reductase), ANS (anthocyanidin synthase), UFGT (UDP-glucose: flavonoid 3-O-glucosyltransferase), and GST (glutathione S-transferase).

Anthocyanins are flavonoid derivatives characterized by a 3,5,7-trihydroxy-2-phenylchromenylium core, with structural diversity arising from hydroxylation patterns on the B-ring [11]. Our transcriptomic analysis revealed that blue:red (5:1) light treatment significantly altered the expression of metabolic genes, particularly in biological processes (BPs) related to primary and secondary metabolism (Figure 4). Notably, the upregulation of molecular function (MF) terms associated with catalytic activity (e.g., GO:0003824) correlated with enhanced enzymatic capacity for anthocyanin biosynthesis. Consistent with photomorphogenic responses in light-sensitive plants [12,13], our findings demonstrate blue light-mediated upregulation of anthocyanin pathway genes (e.g., DFR, ANS) and glutathione S-transferase (GST) activity, mirroring observations in *Arabidopsis* and grape skin [14,15]. This light-responsive GST expression facilitates anthocyanin transport to vacuoles, accounting for the observed pigmentation changes.

### 2.4. Expression Analysis of Enzymes Related to the Biosynthetic Pathway of Anthocyanins

Anthocyanin biosynthesis is an important branch of flavonoid biosynthesis. Although the types of anthocyanins vary among different species, leading to distinct accumulation patterns, their synthetic pathways are essentially the same. According to Chaves-Silva et al. [16], the anthocyanin biosynthetic pathway can be categorized into five distinct stages, each controlled by specific enzymes.

In Stage 1, phenylalanine is converted into 4-coumaroyl-CoA by PAL (phenylalanine ammonia-lyase), C4H (cinnamate 4-hydroxylase), and 4CL (4-coumaroyl-CoA ligase). PAL, the primary rate-limiting enzyme, correlates with anthocyanin accumulation and other phenolic compounds in fruit tissues [17]. C4H is a key catalytic enzyme in the early steps of the phenylpropanoid pathway and one of the most characteristic cytochrome P450 hydroxylases in higher plants [18]. In black raspberry fruit development, the expression of *RsC4H* peaked during early fruit development and red coloration, coinciding with changes in flavonoid content [19]. 4CL plays a pivotal role in the phenylpropanoid pathway by catalyzing the formation of 4-coumaroyl-CoA, a central intermediate that serves as a precursor for both lignin and flavonoid biosynthesis [20]. Under a blue:red (5:1) light ratio, differential gene expression analysis revealed five genes for *PAL*, four for *C4H*, and three for *4CL*. Following the formation of 4-coumaroyl-CoA, Stage 2 involves its conversion into dihydroflavonols through the action of CHS, CHI, and F3H (flavanone 3-hydroxylase).

In Stage 2, 4-coumaroyl-CoA and malonyl-CoA are converted into dihydroflavonols, a process regulated by CHS (chalcone synthase), CHI (chalcone isomerase), and *F3H*. Chalcones are present in trace amounts in plants and serve as anthocyanin precursors [21]. In *Petunia hybrida*, spatial inhibition of CHS resulted in star-shaped pigmentation and edge-specific color changes [22]. Under the blue:red (5:1) light treatment, two differentially expressed *CHS* genes were identified. 

Stage 3 involves the conversion of dihydroflavonols into colorless basic anthocyanidins, regulated by F3′H (flavonoid 3′-hydroxylase), F3′5′H (flavonoid 3′,5′-hydroxylase), DFR (dihydroflavonol 4-reductase), and ANS (anthocyanidin synthase). ANS catalyzes the final step of flavonoid biosynthesis, converting colorless leucoanthocyanidins into colored anthocyanidins [23]. In grape and strawberry, reduced *ANS* expression decreases anthocyanin biosynthesis and lighter organ colors [24,25]. Under the blue:red (5:1) light treatment, one differentially expressed *ANS* gene was identified. 

In Stage 4, unstable anthocyanidins undergo glycosylation to form colored anthocyanins, which may be further modified by methylation, acetylation, or hydroxylation to produce diverse anthocyanin types. UFGT (UDP-glucose: flavonoid 3-*O*-glucosyltransferase), a member of glycosyltransferase family 1, mediates anthocyanin glycosylation [26]. Glycosylation represents the final step in plant flavonoid biosynthesis and signals anthocyanin transport to the vacuole [27]. After the blue:red (5:1) light treatment, nine genes related to glycosylation modification enzymes were identified.

Stage 5 involves anthocyanin synthesis and modification in the cytoplasm and endoplasmic reticulum membrane, followed by vacuolar storage via GST (glutathione *S*-transferase)-mediated transport [28]. Under the blue:red (5:1) light treatment, 23 *GST* genes were differentially expressed, suggesting enhanced anthocyanin transport.

In summary, the blue:red (5:1) light ratio promotes anthocyanin accumulation in *A. melanocarpa* callus by upregulating genes associated with key biosynthetic enzymes (PAL, C4H, 4CL, CHS, ANS, UFGT, and 5AT) and transport-related proteins (GST). These findings highlight the critical role of light quality in modulating anthocyanin transport and vacuolar storage at the cellular level.

### 2.5. Differential Gene qRT-PCR Validation

**Figure 5:**
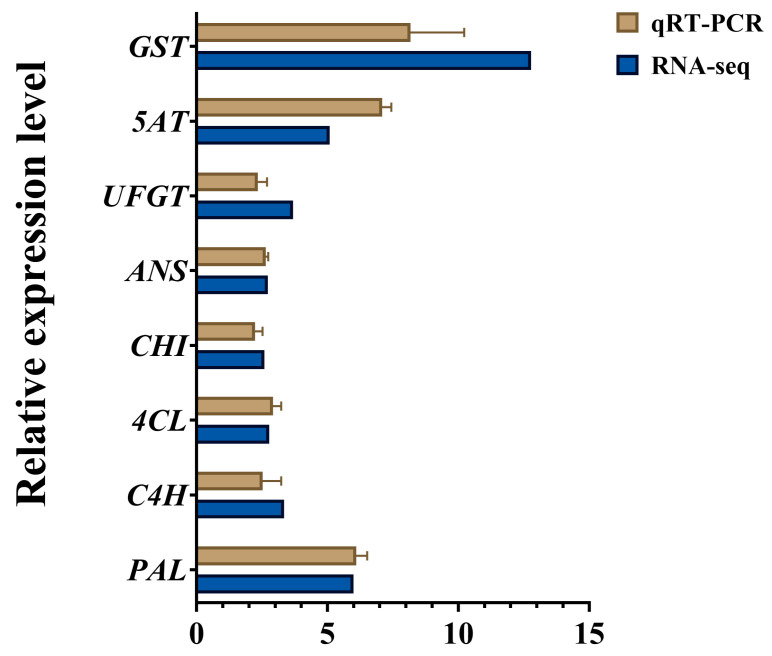
qRT-PCR validation of differentially expressed genes related to anthocyanin synthesis. The relative expression of eight genes from RNA-seq and qRT-PCR in a comparison between blue:red (5:1) and CK. The data are means ± SDs from three biological replicates. Abbreviations: PAL, phenylalanine ammonia-lyase; C4H, cinnamate 4-hydroxylase; 4CL, 4-coumaroyl-CoA ligase; CHI, chalcone isomerase; ANS, anthocyanidin synthase; UFGT, UDP-glucose: flavonoid 3-O-glucosyltransferase; 5AT, anthocyanin 5-aromatic acyltransferase; GST, glutathione S-transferase.

To validate the upregulation of eight DEGs (*PAL*, *C4H*, *4CL*, *CHI*, *ANS*, *UFGT*, *5AT*, and *GST*) from the transcriptome data, we performed qRT-PCR with GAPDH as the reference gene (primer sequences in Table S1). Figure 5 shows that the expression patterns of these genes in the callus under blue:red (5:1) light treatment correlated well with the RNA-seq data. The strong correlation confirms the transcriptome data’s reliability, enabling further mechanistic studies on anthocyanin accumulation regulation in this system.

## 3. Discussion

This study provides transcriptomic insights into anthocyanin biosynthesis under blue:red (5:1) light. While research on *A. melanocarpa* fruits and sterile seedlings has progressed, callus tissue mechanisms remain less explored. Our findings establish light-quality-dependent accumulation patterns and mechanistic pathways, supporting optimized anthocyanin production from stem cell cultures.

Light regulates plant development through its intensity, spectral quality, direction, and photoperiod by modulating enzyme-related gene expression, thereby influencing anthocyanin synthesis [29]. In this study, a blue:red (5:1) light ratio upregulated key anthocyanin biosynthesis genes (*PAL*, *C4H*, *4CL*, *CHS*, *ANS*, *UFGT*, *5AT*, and *GST*) in *A. melanocarpa* callus in vitro, enhancing both synthesis and transport. BP analysis further revealed upregulated responses to abiotic stress, consistent with prior reports that *ANS* expression is induced by light, low temperature, drought, and sugars [30,31,32], and that sucrose transporters in *Arabidopsis* respond to similar cues [33]. Blue light also activates anthocyanin structural gene promoters in pear and strawberry [34,35], while *ANS* is critical for underscoring light’s essential role in fruit coloration [36].

As both an energy source and potential stressor, it triggered photoprotective anthocyanin accumulation in surface-layer callus cells under blue:red (5:1) light [37]. Current evidence suggests that four protein classes (*GST*, *MRP*, *MATE*, and *BTL*-homologues) mediate vacuolar transport [38]. Post-synthesis, *GST* catalyzes glutathione conjugation for membrane trafficking [39], followed by endoplasmic reticulum-to-vacuole transport via transmembrane mechanisms or vesicle uptake [40,41]. These processes align with our CC enrichment analysis, which highlighted plasma membrane and transferase activity.

Despite the valuable insights gained in this study, some limitations should be acknowledged. First, the experiments were conducted in vitro using callus tissues, which may not fully replicate the regulatory complexity of intact plants under natural growth conditions. Second, the light treatments were relatively short-term and thus may not reflect the dynamic and long-term responses of anthocyanin metabolism in field environments. In future studies, functional validation of key candidate genes through gene silencing or overexpression, combined with long-term cultivation experiments or field trials, will be necessary to better assess the stability and practical applicability of these findings. Such work will help bridge the gap between controlled laboratory systems and real-world applications, ultimately facilitating the development of optimized strategies for anthocyanin production in crops and cell cultures.

## 4. Materials and Methods

### 4.1. Plant Material

Calli of *A. melanocarpa* were cultured in the Laboratory of Rare and Endangered Medicinal Plant Cell Engineering at Dalian Polytechnic University. The culture medium consisted of MS basal medium supplemented with 1.5 mg/L NAA, 1.5 mg/L 6-BA, 1.0 mg/L KT, 3.5% (*w*/*v*) sucrose, and 0.7% (*w*/*v*) agarose.

### 4.2. Callus Culture and Anthocyanin Extraction

Under sterile conditions, *A. melanocarpa* calli were sectioned into fragments of approximately 0.2 g and transferred into culture bottles (seven fragments per bottle). The calli were first maintained in darkness for 20 days to promote stable proliferation and to eliminate pre-existing photomorphogenic effects. Subsequently, the cultures were exposed to six different light regimes for 10 consecutive days under a 12 h/12 h light–dark photoperiod. The treatments included (i) full-spectrum LED, (ii) blue:red (5:1), (iii) red:blue (5:1), (iv) red:blue:white (1:1:1), (v) red:white (1:5), and (vi) monochromatic blue light. These spectral combinations were chosen to systematically evaluate the effect of light balance on anthocyanin accumulation. The blue and red LEDs used in this study had peak wavelengths of approximately 450 nm (blue) and 660 nm (red), respectively.

After treatment, the callus tissues were oven-dried at 40 °C to constant weight and ground into a fine powder. Anthocyanins were extracted following a modified protocol of Cissé et al. [42]. Briefly, homogenized powder was mixed with 60% ethanol (1:30, *w*/*v*), and the pH was adjusted to 2.0. Ultrasonic extraction was conducted at 40 °C for 50 min to enhance pigment release, followed by centrifugation at 8000 rpm for 10 min at 4 °C. The supernatant was collected as the crude anthocyanin extract.

Spectrophotometric quantification of anthocyanins was performed using the pH differential method [43]. A 1 mL volume of the extract was diluted to 5 mL with pH 1.0 and pH 4.5 buffers, and equilibrated at 4 °C for 1 h, and then, the absorbance was measured at 510 nm and 700 nm. The anthocyanin content (*W*, mg/g) was calculated using the following Equation (1):

*W* = (*A* × *M* × *DF* × *V*)/(*ε* × *L* × *m*) (1)

where

*A* = Δ(*A*510−*A*700) (pH 1.0) − Δ(*A*510−*A*700) (pH 4.5);
*M* = molecular weight of cyanidin-3-*O*-glucoside (449.2 g/mol);
*DF* = dilution factor;
*V* = final volume (mL);
*ε* = molar absorptivity (26,900 L·mol^−1^·cm^−1^);
*L* = path length (1 cm);
*m* = sample mass (g).

All the quantification steps were conducted at 4 °C to minimize anthocyanin degradation and ensure pigment stability. Statistical analysis was performed using one-way ANOVA to compare the anthocyanin content and growth parameters among treatments, followed by Tukey’s multiple comparison test to identify significant differences. A significance threshold of *p* < 0.05 was applied in all cases.

### 4.3. Transcriptome Sequencing Analysis

Callus samples from the light-optimized group (A, highest anthocyanin content) and dark-cultured control group (B) were collected, each with three biological replicates. Total RNA was extracted with the RNAprep Pure Plant Kit (Tiangen Biotech, Beijing, China), and the RNA quality was confirmed using NanoDrop (Thermo Fisher Scientific, Wilmington, DE, USA)and an Agilent 2100 Bioanalyzer (Agilent Technologies, Santa Clara, CA, USA). RNA-seq libraries were prepared with the NEBNext^®^ Ultra™ RNA Library Prep Kit (New England Biolabs, Ipswich, MA, USA) and sequenced on an Illumina HiSeq 4000 platform (Illumina, San Diego, CA, USA).

De novo transcriptome assembly was conducted using Trinity v2.13.2 [44]. Clean reads were mapped back to the assembled transcriptome using Bowtie2 v2.4.3 [45], and expression levels were quantified with RSEM as TPM and FPKM values. Differentially expressed genes (DEGs) between Groups A and B were identified with DESeq2 v1.38.3 [46] (|log2FC| ≥ 1.5, FDR < 0.05). Functional annotation of unigenes was performed against the NR, Swiss-Prot, Pfam, GO, and KEGG databases (E-value ≤ 1 ✕ 10^−5^). The GO and KEGG enrichment of DEGs were analyzed using topGO [47] and clusterProfiler [48], respectively, with adjusted *p* < 0.05 considered significant [46].

### 4.4. Validation of Differential Gene Expression in the Transcriptome

To validate the transcriptomic results, total RNA from Groups A and B was extracted using the RNAprep Pure Plant Kit (Tiangen Biotech, Beijing, China). The RNA integrity was assessed via agarose gel electrophoresis and Agilent 2100 Bioanalyzer (Agilent Technologies, Santa Clara, CA, USA) to ensure high quality. First-strand cDNA was synthesized using the TransScript SuperMix Kit (TransGen Biotech, Beijing, China). Quantitative real-time PCR (qRT-PCR) was performed with SYBR Green chemistry (Applied Biosystems, Foster City, CA, USA), with GAPDH serving as the reference gene. Primer specificity was confirmed by melt-curve analysis. The relative expression levels of eight candidate DEGs (*PAL*, *C4H*, *4CL*, *CHI*, *ANS*, *UFGT*, *5AT*, and *GST*) were calculated using the 2^−ΔΔCt^ method, based on at least three biological and three technical replicates per sample. The results are presented as mean ± standard deviation, confirming consistency between the qRT-PCR and RNA-seq data.

## 5. Conclusions

In conclusion, our study demonstrates that anthocyanin biosynthesis and accumulation in *A. melanocarpa* callus are strongly influenced by environmental light quality. Among the tested conditions, a blue:red (5:1) light ratio was most effective, resulting in the highest anthocyanin content (14.06 mg/100 g). Transcriptomic analysis revealed that this light regime significantly upregulated key structural and regulatory genes in the anthocyanin biosynthetic pathway, including *PAL*, *C4H*, *4CL*, *CHS*, *ANS*, *UFGT*, and *GST*, thereby enhancing both synthesis and vacuolar transport. These findings provide new transcriptomic insights into how light regulates secondary metabolism in callus tissue, highlighting the importance of light quality in modulating metabolic processes.

Overall, this work establishes *A. melanocarpa* callus as a promising in vitro model for studying anthocyanin biosynthesis and light response. Beyond advancing basic understanding, our results offer a foundation for optimizing cell culture systems for sustainable and high-value anthocyanin production. Future studies involving the functional validation of candidate genes and long-term cultivation trials will further strengthen the application potential of this system.

## Figures and Tables

**Table 1:** Quality evaluation of sequencing data.

Samples	Raw Reads	Clean Reads	Q30 (%)	Error Rate (%)	Effective Sequence Proportion (%)	GC Content (%)
A1	51,461,168	49,428,688	96.54	0.00	96.05	49.96
A2	53,531,486	51,296,672	96.73	0.00	95.82	50.18
A3	50,520,728	48,525,172	96.71	0.00	96.05	49.64
B1	36,101,876	33,998,846	95.66	0.01	94.17	50.19
B2	47,294,422	44,749,004	95.79	0.01	94.61	49.80
B3	57,760,972	55,419,198	96.59	0.00	95.95	49.62

Q30, percentage of bases with Phred quality score ≥30; GC, guanine–cytosine content.

## Data Availability

The raw transcriptome sequencing data used in this study have been deposited in CNGBdb under the accession number CNP0007963. The datasets used and/or analyzed during the current study are available from the corresponding author upon reasonable request.

## Supplementary Materials

The following supporting information can be downloaded at: https://www.mdpi.com/article/10.3390/ijms26199588/s1.
